# Reply

**Published:** 2010

**Authors:** Marzouqah Alanazi

**Affiliations:** Department of Emergency Medicine, King Abdulaziz Medical City, National Guard Health Affairs, Riyadh, Saudi Arabia

We agree that the intramuscular route is superior to the subcutaneous in terms of better absorption and we would like to thank Dr. Gazlan for his comment.

